# The Effect of Chinese Medicinal Formulas on Biomarkers of Oxidative Stress in STZ-Induced Diabetic Kidney Disease Rats: A Meta-Analysis and Systematic Review

**DOI:** 10.3389/fmed.2022.848432

**Published:** 2022-04-15

**Authors:** Qian Zhou, Chuyi Han, Yanmei Wang, Shunlian Fu, Yiding Chen, Qiu Chen

**Affiliations:** ^1^Hospital of Chengdu University of Traditional Chinese Medicine, Chengdu, China; ^2^Research Centre of Pharmaceutical Preparations and Nanomedicine, College of Pharmacy, Chongqing Medical University, Chongqing, China

**Keywords:** diabetic kidney disease (DKD), oxidative stress, Chinese herbal formulas, glutathione peroxidase, superoxide dismutase, malondialdehyde

## Abstract

**Background:**

Diabetic kidney disease (DKD), defined broadly as persistent proteinuria with low estimated glomerular filtration rate in patients with diabetes, is a main cause of end-stage renal disease. Excessive production of reactive oxygen species is an important mechanism underlying the pathogenesis of DKD and many antioxidants have been investigated as therapeutic agents. Among them, Chinese medicine antioxidative stress therapies have been widely used to combat DKD, which may offer new insights into therapeutic development of DKD. There are several discrepancies among the efficacy of Western medicine (WM) and Chinese medicinal formula (CMF) action.

**Methods:**

We searched PubMed, Cochrane Library, the Web of Science databases, Embase, and Scopus from inception to December 2021 using relevant keywords and a comprehensive search for randomized controlled trials (RCTs) was performed. Calculating the pooled weighted mean difference (MD) and *95*% CI by the method of inverse-variance with a random-effect. All the related statistical analyses were performed using Stata version 15.1 software (Stata Corporation) and Rvman version 5.3 (Nordic Cochrane Center).

**Results:**

A total of 8 articles with the 9 groups including 106 in the model group, 105 in the CMF group, and 99 in the WM group. Pooled data from 8 studies (9 groups) showed a statistical improvement in superoxide dismutase compared with the model group [standardized MD (SMD) = 1.57; 95 CI: 1.16–1.98; *P* < 0.05] and the WM group (SMD = 0.56; 95 CI: 0.19–0.92; *P* < 0.05). For glutathione peroxidase (GSH-Px), it was significantly improved in the CMF group vs. the model group and the WM group. For malondialdehyde (MDA), it was significantly reduced in the CMF group (CMF vs. model group: SMD = −1.52; 95 CI: −1.88 −1.17; *P* < 0.05; CMF vs. WM group: SMD = −0.64; 95 CI: −0.95 −0.33; *P* < 0.05).

**Conclusion:**

This systematic review and meta-analysis have demonstrated that the therapy of CMF had a notable curative effect on relieving oxidative stress in STZ-induced DKD rats and CMF was significantly more effective than the WM control group. For the clinical application, the results providing confidence and some theoretical reference for DKD *via* evaluating the efficacy of CMF to a certain extent.

**Systematic Review Registration:**

[PROSPERO], identifier [CRD42022313737].

## Introduction

Diabetic kidney disease (DKD) clinically manifests as persistent microalbuminuria, glomerulosclerosis with low estimated glomerular filtration rate (eGFR), and multiple renal pathological injuries in patients with diabetes (1), which results in end-stage renal disease (ESRD) requiring chronic dialysis or transplantation (2, 3). This population are at high risk of cardiovascular disease and other sequelae of chronic kidney disease mortality (2, 3). Provision of adequate kidney care for patients with chronic kidney disease is costly and requires extensive resources (4). But, the pathogenesis of DKD is still controversial; possibilities for these clinical manifests include treated effectively with renin-angiotensin system inhibition, cholesterol microemboli, hypertensive nephrosclerosis, tubulointerstitial fibrosis, and renovascular disease (5). For the etiology, excessive production of reactive oxygen species (ROS) resulting in oxidative stress (6), which is an important mechanism underlying the pathogenesis attributed to the disturbance of various cellular stress responses (7). In addition, experimental and clinical studies suggest an association between hyperglycemia, oxidative stress, and diabetic complications (8–11). The oxidative stress state, resulting from over increasing free radical production in hyperglycemia, has been considered as a central mediator in the pathogenesis of DKD (9) and its progression to ESRD (12, 13). Currently, the possibility of improving patient outcome by therapeutic interventions aimed at reducing oxidative stress is to the fore (14), such as metformin attenuates streptozotocin (STZ)-induced diabetic nephropathy in rats through the modulation of oxidative stress genes expression (15). At present, Western medicine (WM) has been extensively applied in DKD treatment focusing on a single site or pathway, which does not achieve satisfactory therapeutic results.

Chinese medicinal formula (CMF), composed of different natural product components, has already been used for various chronic diseases and widely emphasized to identify bioactive compounds and molecular mechanisms of renoprotection effects, which possesses promising clinical benefits as primary or alternative therapies for DKD treatment due to the synergistic interactions of multitargets (16). The signaling pathways of CMF therapeutic agents including metabolism regulation, antioxidation, anti-inflammation, antifibrosis, and podocyte protection have been identified as crucial mechanisms in the treatment of DKD (16–18). Studies verified that using Danggui-Shaoyao-San (19) and Puerarin (20) as antioxidants, they had antioxidation property on advanced glycation end products (AGEs) mediated renal injury in STZ-DKD rats by elevating the expression of superoxide dismutase (SOD), malondialdehyde (MDA), and glutathione peroxidase (GSH-Px), regulating the balance of oxidative stress indicators. But, there are several discrepancies among the efficacy of WM and CMF.

In this meta-analysis, we aim to evaluate the effectiveness of CMF on oxidative stress markers in STZ-induced experimental DKD rats and comparing with WM to analyze the mechanistic differences in DKD treatment between Chinese and WM action. The advances in this meta-analysis regarding the multiscale antioxidative stress mechanisms of CMF to effectively reduce DKD, which will offer new insights into therapeutic and alternative therapies in the pathogenesis of DKD and enhance the clinical application of CMF.

## Methods

The Preferred Reporting Items for Systematic Reviews and Meta-Analysis Protocols (PRISMA-P) was followed to perform systematic review (21). Methods will be designed based on the PRISMA (22); the guidelines for reporting were proposed by the Meta-Analysis of Observational Studies in Epidemiology Group (23) and the Cochrane Collaboration Handbook.

### Patient and Public Involvement

Patients or public was not involved in the design, conduct, reporting, or dissemination plans of this research.

Objective: (1) Elucidating the ROS as a mechanism of underlying the pathogenesis of DKD; (2) Exploring the specific mechanism of CMF on antioxidative stress therapies for the treatment of DKD; and (3) Analyzing the mechanistic differences in DKD treatment between Chinese and WM action.

### Search Strategies

A search was performed for literature published from inception to December 2021 using the following electronic bibliographic databases: PubMed, Cochrane Library, the Web of Science databases, Embase, and Scopus. The related terms in the search strategies included “diabetic nephropathies (DNs)” and “diabetic kidney diseases (DKDs)” for the population; the terms including “oxidative stress” were used for exposures and “Chinese medicinal formula (CMF)” was used for interventions. A combination of free terms and controlled vocabulary terms (where applicable) was used in addition to being searched as keywords to ensure that all the relevant studies were identified. Search related literature results were imported into EndNote (version X9).

### Literature Search

We utilized the PRISMA-ScR checklist for reporting scoping reviews of the following article by the inclusion and exclusion criteria.

The literature inclusion criteria are as follows:

(1) Participants: The STZ-induced DKD rat model (without regard to sex or weight).

(2) Interventions: CMFs [formulas composed of traditional Chinese medicine (TCM)] were given by gavage as the CMF groups and no restriction regarding protocol of administration, dose, frequency, or duration.

(3) Comparison: The treatment modality of DKD rats with WM treated as the WM groups and vehicle treated, sham treated (water or saline), and no treatment as the model groups.

(4) Outcomes: Renal oxidative stress indexes: SOD, GSH-Px, and MDA; renal biochemical indexes: fasting blood glucose (FBG) and hemoglobin A1c (HbA1c); and renal function indexes: serum creatinine (Scr), blood urea nitrogen (BUN), urine protein (UP), albumin-creatinine ratio (ACR), and urinary albumin excretion (UAE).

The literature exclusion criteria are as follows:

(1) Participants: The research subject was non-DKD and the research object was human or cells or other animal models.

(2) Interventions: The model groups with integrated CMF and WM, CMF was non-gastric administration, or the experimental group was not CMF treatment.

(3) Comparison: The experimental group was not CMF treatment or integrated WM.

(4) Outcomes: Except all the items mentioned above.

Restricting the search strategy to randomized controlled trials (RCTs) published in English, whereas review, crossover study, meta-analysis, case reports, commentary, clinical trial, editorial, duplicate publication, or study without the separate control group was excluded.

### Study Selection

Two researchers independently performed the study selection of included studies. The procedure involved three steps; first, we utilized EndNote to remove exact duplicate literatures; then, detected irrelevant studies, two authors screened all the titles identified through the electronic database search; and finally, two researchers selected studies from the remaining articles through reading all the abstracts and full texts based on the inclusion and exclusion criteria. Any disagreements or uncertainty was resolved by consensus or discussion with a third researcher.

### Data Extraction

Numerical data from included studies were extracted independently by two investigators; if literatures reported exposure in serving size, but did not specify the amount, recommended conversions were used by Adobe Photoshop CS5 according to a study by Haining et al. (24). The following data from each article were extracted independently by two investigators: (I) general characteristics; the first author, publication year, country, CHF, WM, and sample size and (II) outcome indicators of this study; SOD, GSH-Px, MDA, blood glucose, HbA1c, Scr, BUN, UP, ACR, and UAE of standardized mean difference (SMD) with corresponding 95% CIs. Any discrepancies were discussed and resolved with a third investigator. When it was failed to obtain the necessary information from included literatures, we would write to the corresponding authors for additional data.

### Study Quality Assessment

Two reviewers performed independently with SYRCLE's risk of bias (RoB) tool (25) (an adapted version of the Cochrane RoB tool for animal studies) assessing risk of bias and methodological quality for animal studies; any disagreements or uncertainty was resolved by consensus or discussion with a third researcher. In the assessment tool, there were six types of bias: selection bias, performance bias, detection bias, attrition bias, reporting bias, and other; 10 items were as follows: (1) sequence generation; (2) baseline characteristics; (3) allocation concealment; (4) random housing; (5) blinding; (6) random outcome assessment; (7) blinding of outcome assessment; (8) incomplete outcome data; (9) selective reporting; and (10) other sources of bias. In addition, the appendix (Supplementary Table S1) is given for details.

### Strategy for Data Analysis

For the statistical analysis, a random-effects model was used to account for data analysis due to the exploratory nature of animal studies. SMD with 95% CI for continuous outcomes and risk ratio (RR) with 95% CI for dichotomous outcomes were used to estimate the pooled effects. Additional analyses as sensitivity and subgroup analyses were performed for sources of heterogeneity. Cochran's Q statistic was used to account for anticipated heterogeneity and the proportion of the total variation resulted from heterogeneity was quantified via the *I*^2^ statistic (26) with *I*^2^ > 50% and *P* < 0.05 regarded as having potentially important statistical heterogeneity (27). Publication bias assessment with Begg's test (28) and Egger's test (29) was planned when more than 10 studies were retrieved (30) with *P* < 0.05 indicated potential publication bias. All the related statistical analyses were performed using Stata version 15.1 software (Stata Corporation) and Rvman version 5.3 (Nordic Cochrane Center).

## Results

### Literature Screening Results

**Figure 1:**
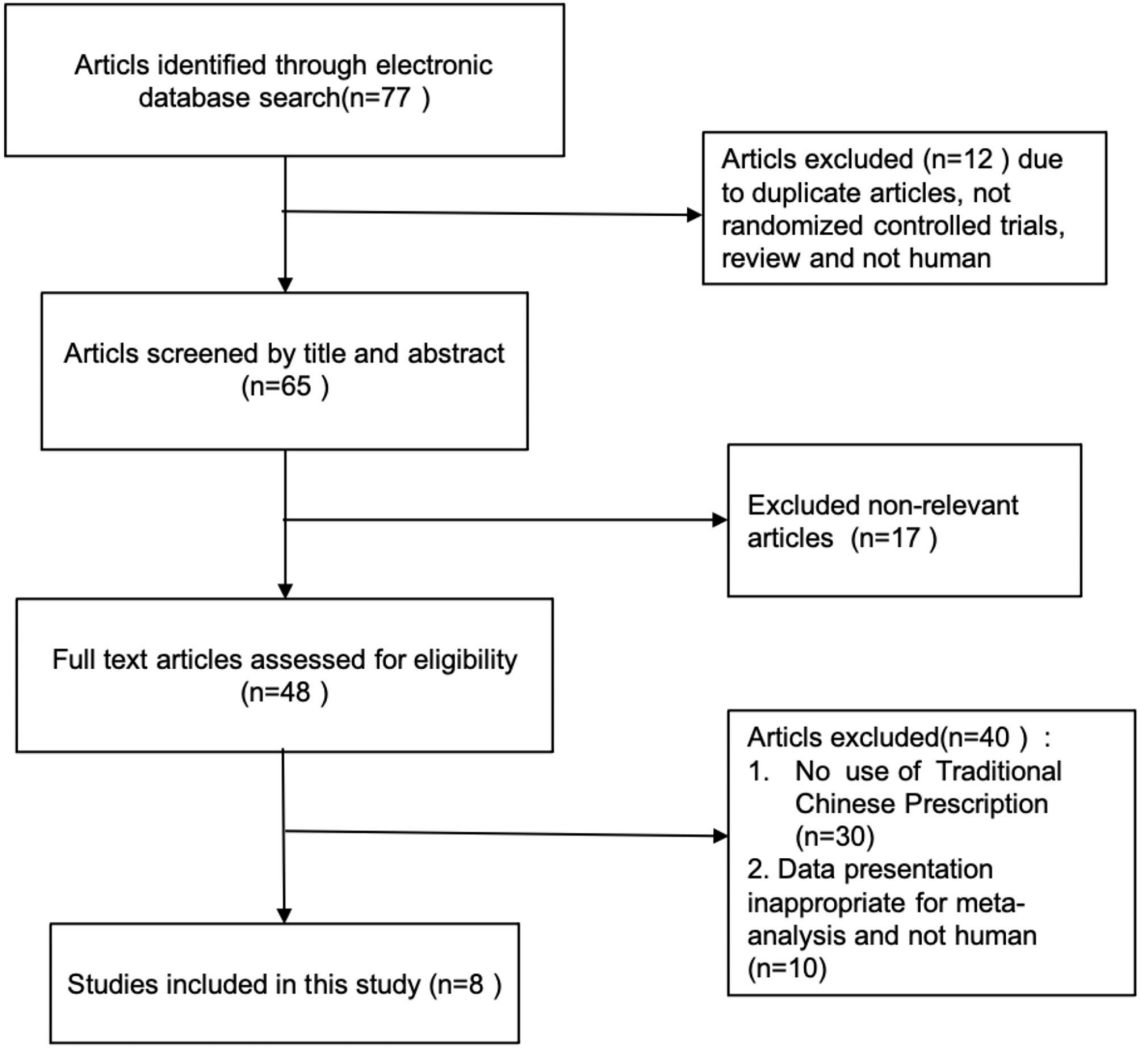
Flowchart of literature search.

A total of 77 citations were initially identified via electronic database searches and the retrieval flowchart is shown in Figure 1. After screening according to the title or abstract, 48 articles were selected for the full-text review. A total of 8 articles met all the inclusion criteria and were included in the meta-analysis after removing irrelevant articles based on exclusion criteria. In Xu's report (31), the subjects of CMF were divided into the Liuwei Dihuang group and the Zhenwu decoction group. Hence, 8 articles (31–38) with the 9 groups were included in the meta-analysis and 84 in the model groups, 96 in the WM groups, and 95 in the CMF groups. The control group was measured including routine treatment and routine treatment combined with WM. The characteristics with more detail of selected studies are given in Table 1.

**Table 1:** Characteristics of included studies.

**Author**	**Published year**	**Country**	**Chinese Medicinal formulas**	**Model group**	**Western medicine**	**Sample size**	**Doses**	**Duration**
						**CMF/Model/WM**		
Zhou et al. (32)	2020	China	Xiaokeyinshuiextract combination	DKD	Metformin	7/7/7	0.5 g/kg	60 days
Fang et al. (35)	2012	China	Fufang xue shuan tong capsules	DKD	captopril	8/7/7	1.8 g/kg	3 months
Li et al. (36)	2021	China	Huayu Tongluo Recipe	DKD	irbesartan	10/9/9	4.59 g/kg	6 weeks
Han et al. (37)	2017	China	Huangqi decoction	DKD	rosiglitazone	8/8/8	0.45 g/kg	8 weeks
Xu et al. (31)	2017	China	Liuwei Dihuang	DKD	valsartan	22/23 /23	6.7 g/kg	8 weeks
			Zhenwu decoction			22/23/23	3 g/kg	8 weeks
Chen et al. (38)	2016	China	Shuangdan Oral Liquid	DKD	Insulin	12/12/12	5 mg/kg	8 weeks
Zhang et al. (33)	2021	China	Yi Shen Pai Du Formula	DKD	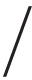	10/10	2.88 g/kg	8 weeks
Zhu et al. (34)	2021	China	Compound centella formula	DKD	losartan	7/7/7	5.4 mg/1kg	80 days

### Quantitative Data Analysis

**Figure 2:**
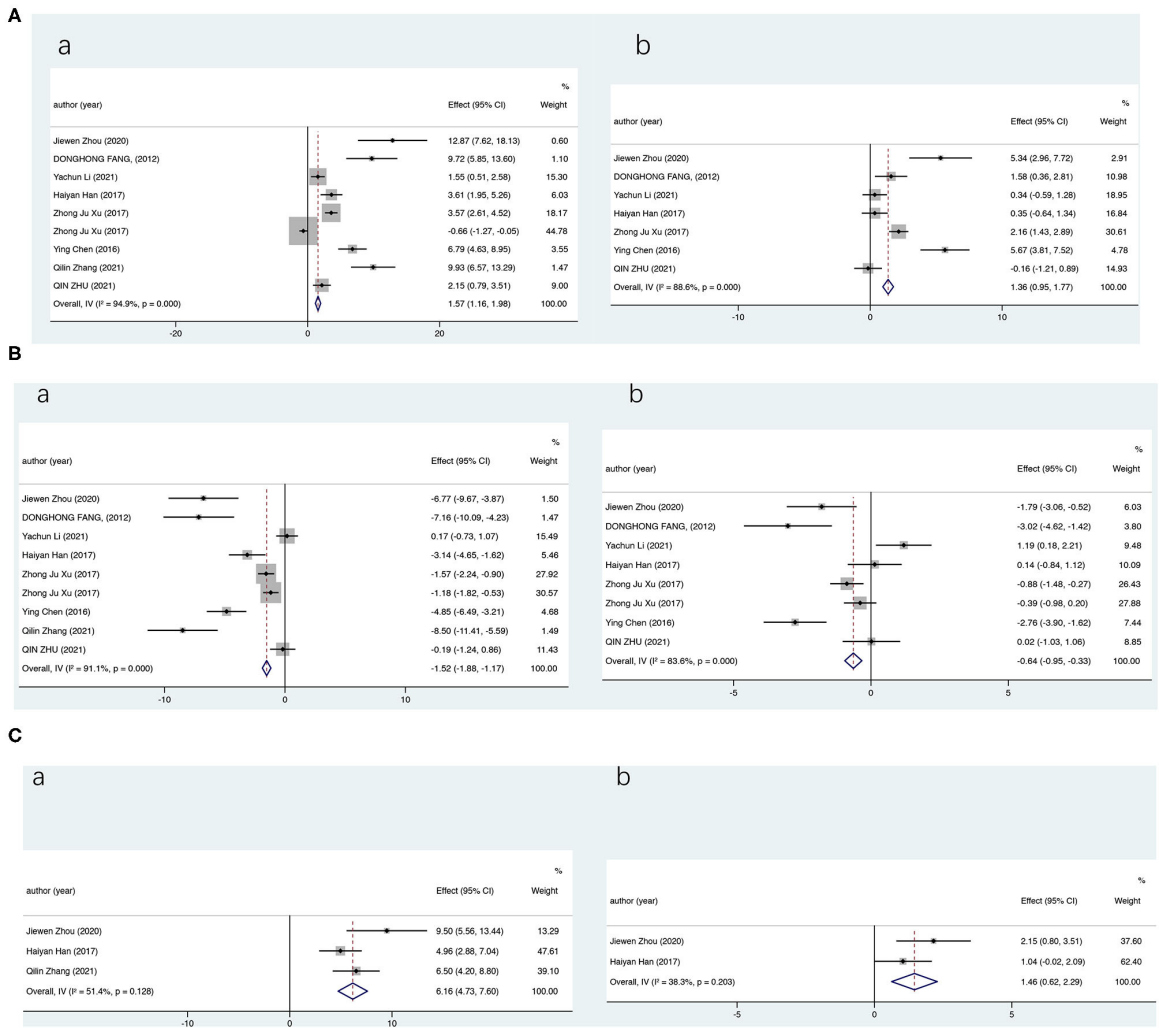
Overall analysis results (CI). Summary estimates were analyzed using a random-effects model. **(A)** For superoxide dismutase (SOD), **(B)** For malondialdehyde (MDA), and **(C)** For glutathione peroxidase (GSH-Px) [(a) vs. the model group and (b) vs. the Western medicine group].

We first evaluate the effects of CMF on the levels of renal oxidative stress indicators in STZ-induced DKD rats and compare them with WM; pooled data from 8 studies (9 groups) showed a statistical improvement in SOD compared with the model group (SMD = 1.57; 95% CI: 1.16–1.98; *P* < 0.000) and the WM group (SMD = 0.56; 95% CI: 0.19–0.92; *P* = 0.003). But, results showed significant heterogeneity by a random-effects model (compared with the model group, *I*^2^ = 94.9%, *P* = 0.000; compared with the WM group, *I*^2^ = 94.5%, *P* = 0.000). The data analysis presented similar results for GSH-PX and significantly improved vs. the model group and the WM group (Figure 2). For MDA, our statistics showed that MDA was significantly reduced in the CMF group (CMF vs. the model group: SMD = −1.52; 95% CI: −1.88 −1.17; *P* < 0.000; CMF vs. the WM group: SMD = −0.64; 95% CI: −0.95 −0.33; *P* < 0.000). The results showed that the therapy of CMF had a significant effect on relieving oxidative stress in DKD and CMF was significantly more effective than WM control. Among the 8 (9 groups) included literatures, the WM groups drugs metformin, insulin, and rosiglitazone were inappropriate and not for the treatment of DKD and one article without positive control. Considering the preciseness of the results, we reperformed data analyses after excluding the four literatures (32, 33, 37, 38) and obtained very similar results (Supplementary Figure S1). Owing to the underrepresentation number of trials, only the SOD and MDA were analyzed.

For glucose, there was a substantially decrease following CMF administration compared with the model group (SMD = −0.726; 95% CI: −1.266 −0.186; *P* < 0.000), but the treatment efficiency was significantly reduced when compared with the WM group (SMD = 1.123; 95% CI: 0.374–1.873; *P* = 0.003). For renal function, the pooled meta-analyses also revealed a statistically significant in Scr (SMD = −1.404; 95% CI: −1.953 −0.856), BUN (SMD = −2.026; 95% CI: −2.569 −1.483), and UP excretion (SMD = −1.370; 95% CI: −2.255 −0.486) and there was no statistical significance between CMF treatment and WM. The detailed results are shown in Table 2.

**Table 2:** Summary of quantitative data analysis.

**Indexes**	**Pooled data**	**Groups**	**Participants**	**Random effects SMD (95% CI)**	**I^2^ (%)**	***P* for heterogeneity**
Superoxide dismutase	vs. model group	9	211	1.57 (1.16, 1.98)	94.90%	*P* < 0.05
	vs. Western medicine group	8	191	0.56 (0.19, 0.92)	94.50%	*P* < 0.05
Malonicaldehyde	vs. model group	9	211	−1.5 (1.88, −1.17)	91.10%	*P* < 0.05
	vs. Western medicine group	8	191	−0.64 (−0.95, −0.33)	83.60%	*P* < 0.05
Glutathione peroxidase	vs. model group	3	50	6.13 (4.74, 7.6)	51.40%	*P* = 0.128
	vs. Western medicine group	2	30	1.46 (0.62, 2.29)	38.30%	*P* = 0.203
Glucose	vs. model group	3	59	−0.73 (−1.27, −0.19)	54%	*P* = 0.114
	vs. Western medicine group	2	38	1.123 (0.374, 1.873)	90.80%	*P* < 0.05
Serumcreatinine	vs. model group	4	77	−1.404 (−1.953, −0.856)	86.20%	*P* < 0.05
	vs. Western medicine group	3	56	0.471 (−0.061, 1.003)	0.00%	*P* = 0.983
Blood urea nitrogen	vs. model group	5	93	−2.026 (−2.569, −1.483)	79.90%	*P* < 0.05
	vs. Western medicine group	4	72	−0.247 (−0.753, 0.260)	85.20%	*P* < 0.05
Urinary protein excretion	vs. model group	2	31	−1.370 (−2.255, −0.486)	90.50%	*P* < 0.05
	vs. Western medicine group	2	30	−0.245 (−0.966, 0.476)	0%	*P* = 0.552

### Subgroup Analyses and Sensitivity Analyses

We found that the heterogeneity was significantly higher than previous comparisons. Subsequently, the subgroup analyses were performed to investigate the factors that influenced heterogeneity. In this meta-analysis, due to the underrepresentation number of trials in some subgroups, neither of these studies completed subgroup analysis. The more detailed analyses were performed in subgroups with representation number of trials. Due to the diversity of interventions, the intervention duration and doses varied considerably. Considering different dosage of CMF used in trials, studies were divided into the two subgroups: the low-dose group (≤1 g/kg/day) and the high-dose group (>1 g/kg/day). Compared with the model group, we found that SOD activity showed significant improved in the low-dose group (SMD = 2.47; 95% CI: 1.49–3.46) and the high-dose group (SMD = −0.29; 95% CI: −0.67 −0.09). The subgroup analyses showed that no significant difference (SMD = 0.27; 95% CI: −0.16–0.71) was identified when the administration of the high-dose CMF groups in comparison with the WM groups, but the low-dose group was significantly different (SMD = 1.17; 95% CI: 0.52–1.81). Regarding duration of intervention, studies were divided into the two subgroups: the intervention interval <8 weeks group and the interval ≥ 8 weeks group. The subgroup analyses presented similar results for duration of the intervention subgroups and significantly improved between the model group and the WM group. But, in the interval ≥ 8 weeks subgroup, no significant difference (SMD = 0.33; 95% CI: −0.10–0.75) was identified compared with the WM group. Considering the variation of MDA in the subgroup analyses, the data analysis results are similar to those of the overall analysis. Except for the intervention interval <8 weeks subgroup, there was no statistically significance between CMF treatment and WM (SMD = −0.57; 95% CI: −1.28–0.14). For specific details, see Table 3.

**Table 3:** Summary of subgroup analysis.

**Oxidative stress indicators**	**Subgroup analysis**	**Groups**	**Participants**	**Random effects SMD (95% CI)**	**I^2^ (%)**	***P* for heterogeneity**
	**vs. model group**					
Super oxide dismutase	low-dose group	4	68	3.80 (2.87, 4.73)	87.80%	*P* < 0.05
	high-dose group	5	143	1.04 (0.59, 1.49)	96.20%	*P* < 0.05
Malonicaldehyde	low-dose group	4	68	−2.26 (−3.00, −1.52)	91.40%	*P* < 0.05
	high-dose group	5	143	−1.30 (−1.71, −0.90)	92.00%	*P* < 0.05
Super oxide dismutase	interval <8 weeks group	3	48	2.47 (1.49, 3.46)	93.70%	*P* < 0.05
	Interval ≥ 8 weeks group	6	163	1.38 (0.94, 1.83)	95.90%	*P* < 0.05
Malonicaldehyde	interval <8 weeks group	3	48	−0.97 (−1.80, −0.15)	94.80%	*P* < 0.05
	Interval ≥ 8 weeks group	6	163	−1.65 (−2.04, −1.26)	89.80%	*P* < 0.05
	vs. Western medicine group					
Super oxide dismutase	low-dose group	4	64	1.17 (0.52, 1.81)	93.10%	*P* < 0.05
	high-dose group	4	127	0.27 (−0.16, 0.71)	96.30%	*P* < 0.05
Malonicaldehyde	low-dose group	4	64	−0.92 (−1.47, −0.37)	84.50%	*P* < 0.05
	high-dose group	4	127	−0.51 (−0.89, −0.13)	83.60%	*P* < 0.05
Super oxide dismutase	interval <8 weeks group	3	46	1.20 (0.49, 1.91)	86.90%	*P* < 0.05
	Interval ≥ 8 weeks group	5	145	0.33 (0.49, 1.91)	96.3%%	*P* < 0.05
Malonicaldehyde	interval <8 weeks group	3	46	−0.57 (−1.28, 0.14)	91.80%	*P* < 0.05
	Interval ≥ 8 weeks group	5	145	−0.66 (−1.01, −0.31)	78.20%	*P* < 0.05

### Publication Bias and Quality Assessment

**Figure 3:**
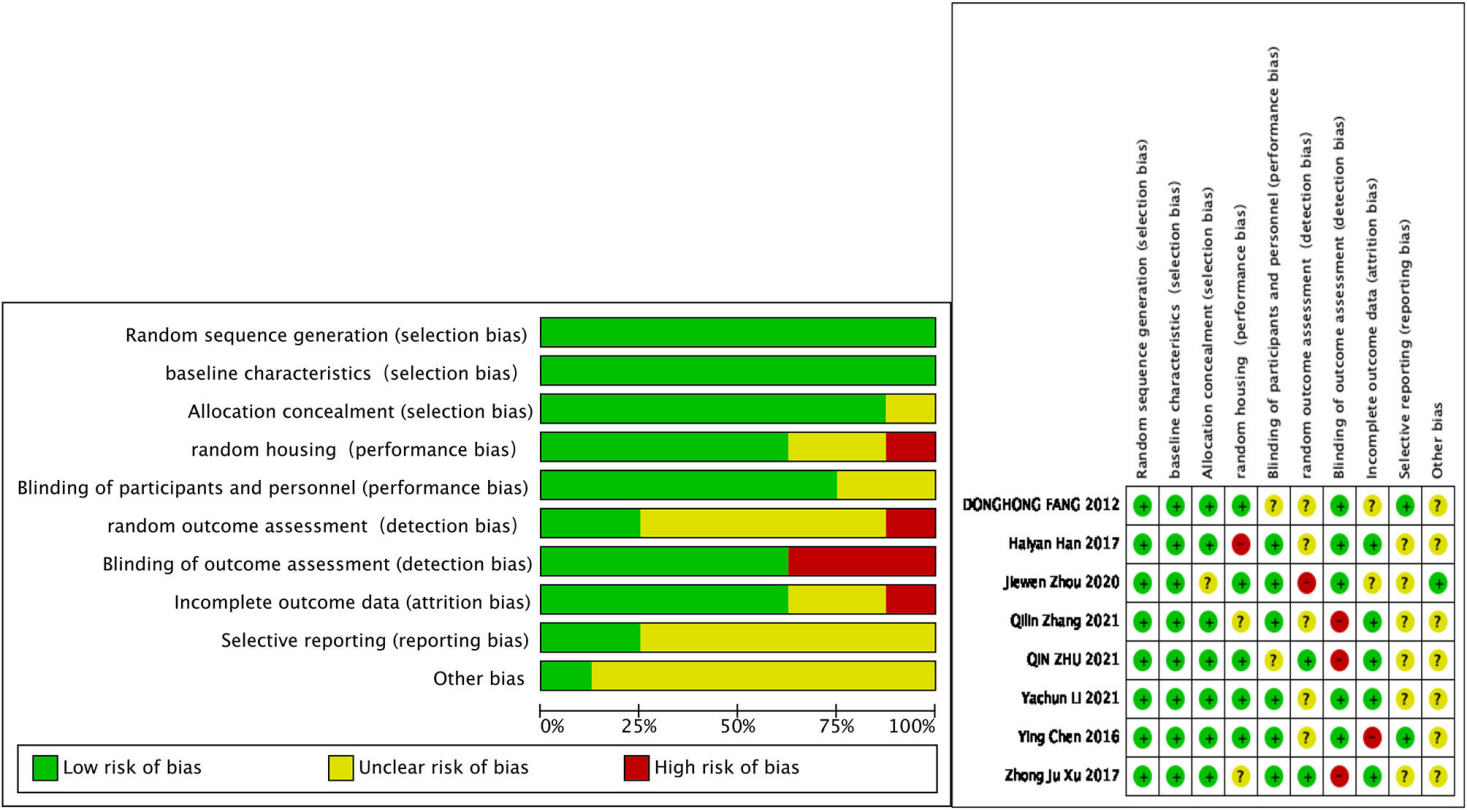
The risk of bias summary of review authors judgments.

The methodological quality and bias of all the eligible studies for animal studies were assessed using SYRCLE's RoB tool. The RoB summary of review authors judgments about each item for each included study was given in Figure 3. Overall, the studies included were of high quality and the quality evaluation of the involved 8 articles was all the randomized, placebo-controlled trials and explains whether their baseline measurements were comparable. For publication bias, there is no need for the diagnostic method from inadequate number of studies (<10).

## Discussion

To the best of our knowledge, this was the first meta-analysis to evaluate the effects of CMF on the levels of kidney oxidative stress indicators in DKD and compared with WM and 8 original studies with the 9 groups were included in this meta-analysis. This meta-analysis has demonstrated that the therapy of CMF had a significant effect on relieving oxidative stress in DKD and more effective than the WM control groups. For renal function, the pooled meta-analyses revealed Scr, BUN, and UP excretion that decreased significantly. Giving additional evidence that effect of alleviating oxidative stress significantly correlated with the intervention duration and doses of CMF. Data provide evidence that CMF treatment of DKD *via* antioxidative stress therapies will improve the value of Chinese medicine in clinical application as well as offering new insights into therapeutic development of DKD.

Oxidative stress is caused by the imbalance between ROS production and the counteracting oxidation mechanisms (39) and the generated highly oxidizing environment refers to the cumulative effects of reactive oxidizing molecules, which cause tissue damage (40). It resulting in impaired critical cellular macromolecules and/or modulate gene expression pathways and producing unwanted modifications to lipids, proteins, DNA, etc. (41, 42). Diabetic has been reported to be closely related to highly oxidative stress *in vivo* and it is the result of the glucose antioxidation, protein glycation, lipid peroxidation, and decrease of antioxidant enzymes activities (43). The results of hyperglycemia-induced secondary mediators activation are responsible for oxidative stress-induced renal injury in the diabetic condition (44). More seriously, oxidative stress can trigger the inflammatory process. Subsequently, local chronic inflammatory stress impairs the antioxidant defense systems, thereby aggravating renal injury vicious cycle (45, 46). Therefore, increased production and/or ineffective scavenging of ROS plays a critical role in certain DKD pathologic state (9) and its progression to ESRD (12, 13) has been suggested to be a underlying pathway linking diverse mechanisms for the pathogenesis of DKD (47). However, ROS is very unstable *in vivo* with very short half-lives, which is difficult to detect directly (48). In contrast, the lifetime of the oxidation products used to assess the redox state varies from hours to weeks (14, 48). The antioxidant enzymes mainly include SOD and GSH-Px, which defense against oxidative stress in the kidney of STZ-induced diabetic rats (49, 50). The most important marker of oxidative stress is MDA, a macromolecular oxidation product that is increased in kidney injured tissues (51).

In this meta-analysis, we analyzed the kidney levels of oxidative stress biomarkers, including SOD, GSH-Px, and MDA. The results found that the CMF group showed a statistical improvement in SOD and GSH-Px when compared with the model group and the WM group. MDA was significantly reduced in the CMF group. Although the results showed that there was little difference in renal function between the CMF group and the WM group, renal function has been significantly improved. The subgroup analyses results showed that the longer and lower dose of CMF was, the better the treatment effects would be. Therefore, the therapeutic efficacy of CMF was remarkable and achieved better efficacy results for antioxidant therapies than WMs. Antioxidant therapies of TCM may be beneficial in reducing oxidative stress and improve renal function and survival. However, heterogeneity exists among the included studies, which may partly originate from the differences in the pooled studies, such as pharmacological differences, animal species, and experimental design. Additional evidences showed that hypoglycemic effect is not always an essential process for the renoprotective effects of TCM and the most effective therapeutic measures must be according to various physiological states need.

The WHO had reported that TCM takes a significant role in maintenance life, treatment, and prevention of some medical conditions and chronic complications of above diseases (52). CMFs are composed of natural products and have been considered as an effective treatment and prevention measure for diabetes (53, 54). At present, WM has been extensively applied in DKD treatment focusing on a single site or pathway, which does not achieve satisfactory therapeutic results. TCM exerting promising therapeutic effect and is even superior to some traditional therapies owing to the multibioactive compounds and molecular targets of action compared to Western agents, with rarely observed adverse reactions in clinical practice (55–57). Therefore, many traditional medicines antioxidants have been demonstrated to provide protection in DKD rats through regulating oxidative stress as therapeutic agents, such as *Angelica* and *Astragalus* (58), resveratrol (59), Huangkui capsule (60), crocin (61), and phillyrin (62). According to TCM theory, TCM experts speculate that the disease location of DKD is kidney and closely associated with the spleen and liver dysfunction (63). The treatment principles of DKD are tonifying Qi and kidney, nourishing yin, removing dampness, resolving phlegm, and blood stasis (63). In terms of mechanisms, studies have reported that molecular targets and signaling pathways to prevent renal injury caused by AGEs accumulation and ROS over production induced by hyperglycemia, involved in the renoprotective effects of TCM, might include insulin receptor substrate/phosphatidylinositol 3-kinase/Akt/glucose transporter type 4 (IRS/PI3K/Akt/GLUT4), NF-E2-related factor 2/antioxidant response element (Nrf2/ARE), nuclear factor-k-gene binding (NF-kB), endothelin-1/endothelin receptors A (ET-1/ETAR), Transforming Growth Factor -β/Smad3 (TGF-β/Smad3), etc. (16, 50, 64–67).

However, the results of this meta-analysis were inconclusive and the conclusion cannot represent the results of clinical trials because of the several limitations of the survey. The studies we cited were the heterogenous group with different preparations of CMF compared to angiotensin-converting enzyme (ACE) inhibitors, angiotensin receptor blockers, metformin, insulin, and rosiglitazone. It is insufficient to show that any one CMF performed well-against a particular WM due to not enough in each group to do the insufficient subgroup analyses. These meta-analysis results were based on rats with chemically-induced diabetes, not obese humans who develop diabetes as a result of poor glucose control. As for the efficacy of TCM, considering the synergistic effects of multi-ingredients and multitherapeutic targets, the use of individual compounds purified or blended bioactive ingredients might be more reliable. In addition, applying grade-/stage-specific strategies as the guide for precise individual therapy in accordance with different grades/stages of DKD, so as to optimize the clinical efficacy of TCM. The mechanisms involved in DKD initiation and progression as well as the renal therapy and protection effect of TCM are not fully elucidated; more well-designed, large-scale RCTs were need to verify the clinical safety and effectiveness of TCM in patients with DKD. In this follow-up study, we will select a combination or single TCM with sufficient clinical evidence and recommended by the guidelines for review to ensure that the bioactive constituents were responsible for the TCM antioxidant.

## Conclusion

The strategy of applying TCM to treat and manage DKD has broad prospects for development, since the significant efficacy and safety in clinical trials can appear to provide therapeutic benefits. The CMF efficiently alleviated oxidative stress via reducing the macromolecule oxidative products (e.g., MDA) and increasing the antioxidant enzymes (e.g., SOD and GSH-Px). Part of the therapeutic mechanism of CMF treatment in STZ-induced DKD rats is attributed to the beneficial antioxidant effects. The relative contribution and relevance of the TCM in the pathogenesis of DKD should be the focus of future studies. Elucidating the cellular and molecular basis of some novel TCM pathways will help us to develop more effective therapies for DKD and also helps us to develop more effective therapies for DKD, which is of great clinical significance for reducing DKD-related morbidity and mortality.

## Data Availability Statement

The original contributions presented in the study are included in the article/Supplementary Material, further inquiries can be directed to the corresponding author.

## Author Contributions

QZ and QC: research idea. QZ and CH: study design and literature search. YW, SF, and YC: data extraction. QZ, YW, and CH: analysis. All authors wrote the manuscript and contributed important intellectual content during manuscript drafting and revision, and accepts accountability for the overall study by ensuring that questions pertaining to the accuracy or integrity of any portion of this study are appropriately investigated and resolved.

## Funding

The study was partly supported by grants from the Chengdu Science and Technology Project (No 2019-YF09-00094-SN) and the Medical Service and Guarantee Capacity Improvement Subsidy Funds (major and difficult diseases-No CYW2019079).

## Conflict of Interest

The authors declare that the research was conducted in the absence of any commercial or financial relationships that could be construed as a potential conflict of interest.

## Publisher's Note

All claims expressed in this article are solely those of the authors and do not necessarily represent those of their affiliated organizations, or those of the publisher, the editors and the reviewers. Any product that may be evaluated in this article, or claim that may be made by its manufacturer, is not guaranteed or endorsed by the publisher.
