# Supplementary material for: The Effect of Chinese Medicinal Formulas on Biomarkers of Oxidative Stress in STZ-Induced Diabetic Kidney Disease Rats: A Meta-Analysis and Systematic Review
Source: Front Med (Lausanne). 2022 Apr 15;9:848432. doi: 10.3389/fmed.2022.848432 (PMC9051386; doi:10.3389/fmed.2022.848432)
Supplement: Supplementary file 1 [file Data_Sheet_1.pdf]

Table S1

SYRCLE's tool for assessing risk of bias

**Item**	**Type of bias**	**Domain**	**Description of domain**	**Review authors judgment**
1	Selection bias	Sequence generation	Describe the methods used, if any, to generate the allocation sequence in sufficient detail to allow an assessment whether it should produce comparable groups.	Was the allocation sequence adequately generated and applied? (*)
2	Selection bias	Baseline characteristics	Describe all the possible prognostic factors or animal characteristics, if any, that are compared in order to judge whether or not intervention and control groups were similar at the start of the experiment.	Were the groups similar at baseline or were they adjusted for confounders in the analysis?
3	Selection bias	Allocation concealment	Describe the method used to conceal the allocation sequence in sufficient detail to determine whether intervention allocations could have been foreseen before or during enrolment.	Was the allocation adequately concealed? (*)

4	Performance bias	Random housing	Describe all measures used, if any, to house the animals randomly within the animal room.	Were the animals randomly housed during the experiment?
5	Performance bias	Blinding	Describe all measures used, if any, to blind trial caregivers and researchers from knowing which intervention each animal received. Provide any information relating to whether the intended blinding was effective.	Were the caregivers and/or investigators blinded from knowledge which intervention each animal received during the experiment?
6	Detection bias	Random outcome assessment	Describe whether or not animals were selected at random for outcome assessment, and which methods to select the animals, if any, were used.	Were animals selected at random for outcome assessment?
7	Detection bias	Blinding	Describe all measures used, if any, to blind outcome assessors from knowing which intervention each animal received. Provide any information relating to whether the intended blinding was effective.	Was the outcome assessor blinded?

8	Attrition bias	Incomplete outcome data	Describe the completeness of outcome data for each main outcome, including attrition and exclusions from the analysis. State whether attrition and exclusions were reported, the numbers in each intervention group (compared with total randomized animals), reasons for attrition or exclusions, and any re-inclusions in analyses for the review.	Were incomplete outcome data adequately addressed? (*)
9	Reporting bias	Selective outcome reporting	State how selective outcome reporting was examined and what was found.	Are reports of the study free of selective outcome reporting? (*)
10	Other	Other sources of bias	State any important concerns about bias not covered by other domains in the tool.	Was the study apparently free of other problems that could result in high risk of bias? (*)

*Items in agreement with the items in the Cochrane Risk of Bias tool(Higgins et al., 2011).

Other biases

The final domain includes other sources of bias. This domain allows review authors to add one or more specific items that address issues particular to their review, and for which the considerations above do not completely cover anticipated risks of bias. For example, some potential biases are relevant only to particular trial designs (e.g. carry-over effects in crossover trials and recruitment bias in cluster-randomized trials);

and there may be sources of bias that are only found in particular clinical settings (e.g. contamination, a form of performance bias in whereby participants experience some or all of an intervention allocated to a different group). Specific items for this domain should preferably be pre-specified in the review protocol, along with a decision as to whether they will be assessed for trials as a whole, or for individual (or grouped) outcomes within each trial.

Items included in this domain should be direct causes of bias, and should not be (i) sources of heterogeneity (e.g. choice of comparator, length of follow-up), (ii) sources of imprecision or over-precision (e.g. failure to account for clustering); or (iii) quality indicators that are not direct causes of bias (e.g. sample size calculations; ethical approval, source of funding).

Figure S1

Overall analysis results. CI, Confidence interval. Summary estimates were analyzed using a random-effects model. A: for SOD, B: for MDA (a VS. model group, b VS. Western medicine group).

A

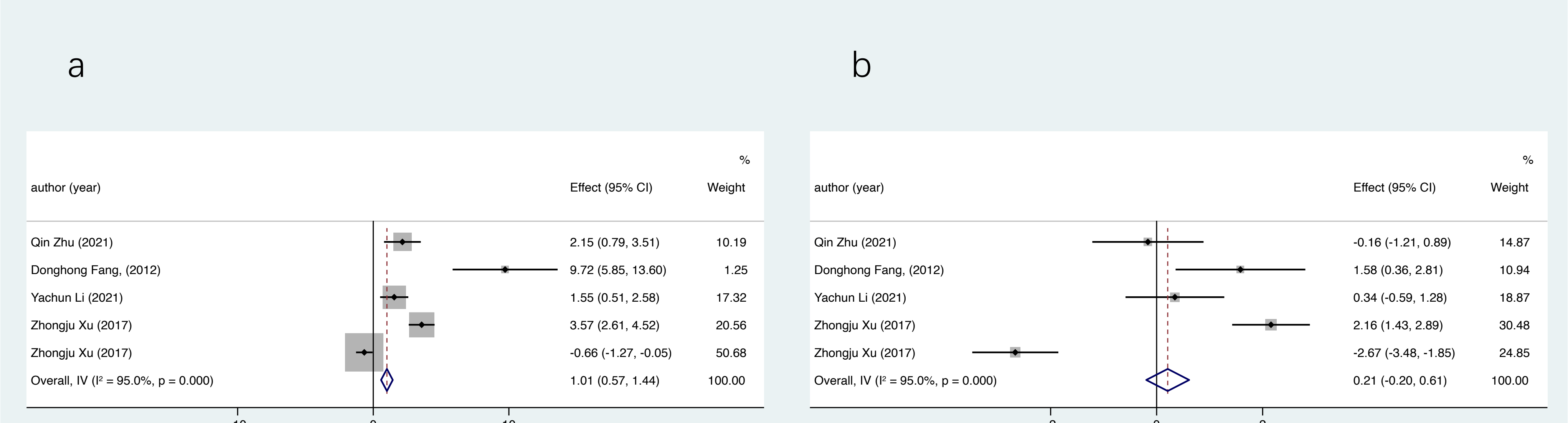

B

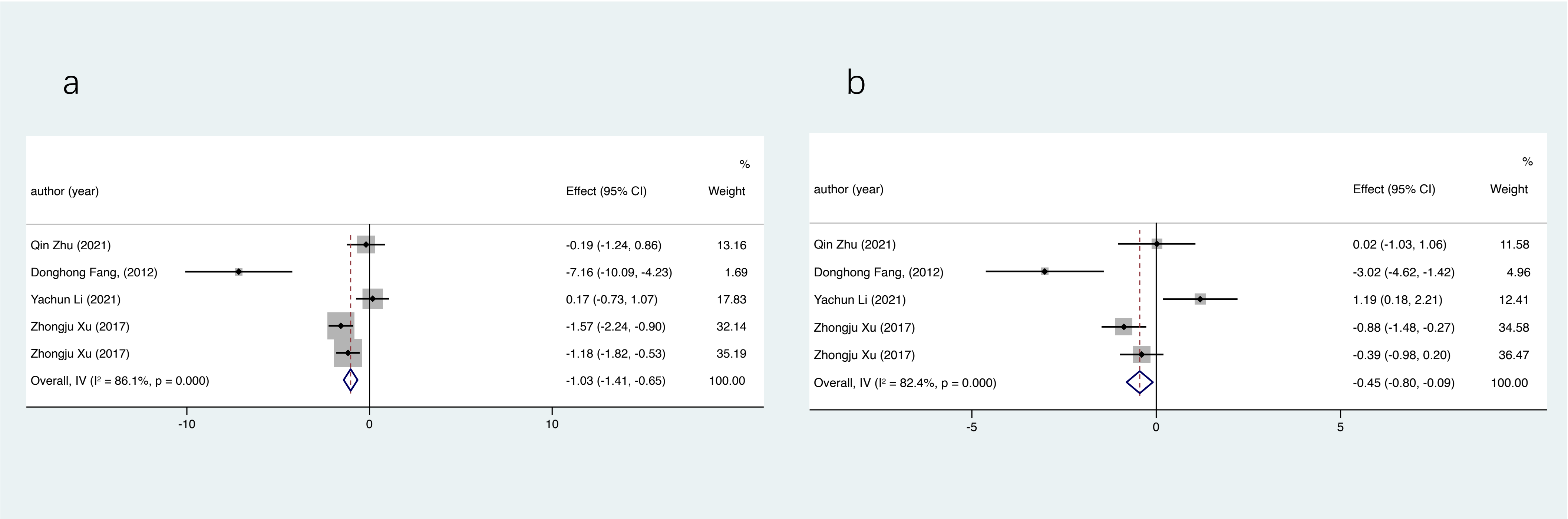

a
author (year)
Effect (95% CI)
% Weight
Qin Zhu (2021)
-0.19 (-1.24, 0.86)
13.16
Donghong Fang, (2012)
-7.16 (-10.09, -4.23)
1.69
Yachun Li (2021)
0.17 (-0.73, 1.07)
17.83
Zhongju Xu (2017)
-1.57 (-2.24, -0.90)
32.14
Zhongju Xu (2017)
-1.18 (-1.82, -0.53)
35.19
Overall, IV (I² = 86.1%, p = 0.000)
-1.03 (-1.41, -0.65)
100.00
-10
0
10
b
author (year)
Effect (95% CI)
% Weight
Qin Zhu (2021)
0.02 (-1.03, 1.06)
11.58
Donghong Fang, (2012)
-3.02 (-4.62, -1.42)
4.96
Yachun Li (2021)
1.19 (0.18, 2.21)
12.41
Zhongju Xu (2017)
-0.88 (-1.48, -0.27)
34.58
Zhongju Xu (2017)
-0.39 (-0.98, 0.20)
36.47
Overall, IV (I² = 82.4%, p = 0.000)
-0.45 (-0.80, -0.09)
100.00
-5
0
5

Higgins, J.P., Altman, D.G., Gøtzsche, P.C., Jüni, P., Moher, D., Oxman, A.D., et al. (2011). The Cochrane Collaboration's tool for assessing risk of bias in randomised trials. *Bmj* 343, d5928. doi: 10.1136/bmj.d5928.
